# The current status of emergency contraception use in reproductive-aged Korean women: a population-based internet survey

**DOI:** 10.3389/fendo.2023.1191096

**Published:** 2023-06-08

**Authors:** You Min Lee, Sung Eun Kim, DooSeok Choi, Dong-Yun Lee

**Affiliations:** Department of Obstetrics and Gynecology, Samsung Medical Center, Sungkyunkwan University School of Medicine, Seoul, Republic of Korea

**Keywords:** emergency contraception, reason, anxiety, counseling, reproductive-aged women, survey

## Abstract

**Objective:**

This study was conducted to assess the current status of emergency contraception (EC) use in reproductive-aged Korean women.

**Materials and methods:**

This study utilized a population-based, cross-sectional online survey using a self-completed questionnaire in women aged 20-44 years who had visited a clinic in the previous six months for contraception counseling. Reason for use, anxiety, and counseling for further contraception at EC use were analyzed according to age, history of childbirth, and contraceptive failure in EC users.

**Results:**

Among 1,011 respondents, 461 (45.6%) had experience with EC use. Younger age, need for EC due to inadequate contraception, and high anxiety were highly prevalent among EC users. However, women in the 20s were less likely to get counseling for further contraception at EC use. Additionally, the proportions of women who used EC due to inadequate contraception during sexual intercourse and who experienced high anxiety were lower among women who had a history of childbirth. Women who had a history of contraceptive failure worried less about EC use.

**Conclusion:**

Our findings offer insight for developing and improving individualized strategies for appropriate contraception, especially in young Korean EC users.

## Introduction

Unwanted pregnancy is an important health, social, and political issue worldwide. Despite recent developments in contraceptive technology, nearly half of all pregnancies in both developed and under-developed countries are estimated to be unintended (1, 2). However, many women who do not desire pregnancy engage in unprotected sexual intercourse (3). Given that many women postpone or avoid pregnancies, more attention should be paid to helping reproductive-aged women choose effective and safe contraception.

Among various contraceptive methods, emergency contraception (EC) is used to prevent pregnancy after sexual intercourse. High doses of combined oral contraceptive were previously used for EC, but this method has been replaced by other hormonal methods such as levonorgestrel or ulipristal acetate because they are more effective and have fewer adverse effects. The copper intrauterine device is also used as an effective and cost-effective alternative (4). Oral EC methods mainly inhibit or delay ovulation, while copper intrauterine device prevents fertilization or implantation rather than ovulation (5). Although EC is effective and safe when used in a timely manner after unprotected sexual intercourse, it is not recommended as a routine contraceptive method in the general population.

Additionally, culture or social attitudes toward contraception can affect perceptions and behaviors related to contraception, and medical environments differ across countries (6).

In these circumstances, it is necessary to develop appropriate and specific strategies regarding EC use that are suitable for each situation, and determining the status of EC use is an important initial step towards this. Nevertheless, studies regarding the current EC status are limited (7, 8), and population-level data are scarce in Korea as well as Asian countries, although population- or culture-based targeted research is essential. Therefore, this population-based, cross-sectional online study was conducted to assess the status of EC use in reproductive-aged Korean women which is relevant to develop individualized strategies for appropriate contraception in EC users.

## Materials and methods

This study analyzed data from the Thinking About Life with Contraception in Korean Women study, which was a quantitative online survey of women and healthcare providers. Study details are described elsewhere (9).

Briefly, a questionnaire for assessing perceptions and behaviors regarding contraception was developed by an advisory board, based on a European study instrument (10). The target population was Korean women aged 20–44 years who had visited a clinic in the previous six months for contraception counseling and who used contraceptives. A research company randomly selected eligible women for participation using stratified random sampling with consideration of the national population statistics for region and age (units of 10 years) from the company’s access panel of 420,000 women. The company sent the questionnaire via URL to avoid duplications. In April 2019, 47,848 women were invited to participate, and 8,177 responded (response rate: 17.1%). Among them, 1,011 women passed the screening process using questions about visiting a clinic for contraception within the past six months and about using any contraceptive method currently, and participated in the survey. Sampling error was calculated as ±3.08% with 95% confidence. Informed consent was obtained electronically from all participants, who were provided with a guide for data protection and personal privacy. All data were gathered and stored anonymously. The study protocol was approved by the Institutional Review Board of Samsung Medical Center.

Among various topics in the questionnaire, questions regarding EC were extracted and analyzed. EC users were defined as women who had ever used oral medications or intrauterine devices for EC, regardless of whether they were used as the primary method of contraception. Age, income, education level, and history of pregnancy, childbirth, or contraception failure were compared between EC users and non-users. The most important source of information about contraception was also compared between the two groups. In addition, reason for use, self-report of anxiety, and counseling for further contraception at the time of EC were analyzed according to age and history of childbirth and contraceptive failure in EC users.

Data are presented as number (percentage). The Chi-square test was used to compare categorical data. P-values less than 0.05 were considered significant. All statistical analyses were performed using SPSS Statistics 27 software (SPSS Inc., Chicago, IL, USA).

## Results

The characteristics of the study participants are presented in **Table 1**. Among the participants, 461 (45.6%) women had experience with EC use. Compared with non-users, EC users were more likely to be younger and less likely to have a history of childbirth. Additionally, EC users were more likely to have a history of contraception failure. However, no difference was found in income, educational level, or history of pregnancy between EC users and non-users.

**Table 1:** Characteristics of study participants.

	EC use (N = 1,011)		*P*-value
	No (N = 550)	Yes (N = 461)	
Age			0.022
20–29 (N=379)	199 (36.2)	180 (39.0)	
30–39 (N=408)	211 (38.4)	197 (42.8)	
40–44 (N=224)	140 (25.4)	84 (18.2)	
Monthly income*			0.982
<200 (1,600 USD) (N=89)	47 (8.5)	42 (9.1)	
200–<500 (N=482)	265 (48.2)	217 (47.0)	
≥500 (4,000 USD) (N=418)	226 (41.1)	192 (41.7)	
Unknown (N=22)	12 (2.2)	10 (2.2)	
Education level			0.131
High school or below (N=113)	69 (12.5)	44 (9.5)	
University or above (N=898)	481 (87.5)	417 (90.5)	
History of pregnancy			0.448
No (N=454)	241 (43.8)	213 (46.2)	
Yes (N=557)	309 (56.2)	248 (53.8)	
History of childbirth			0.032
No (N=522)	267 (48.5)	255 (55.3)	
Yes (N=489)	283 (51.5)	206 (44.7)	
History of contraception failure			0.003
No (N=724)	415 (75.5)	309 (67.0)	
Yes (N=287)	135 (24.5)	152 (33.0)	

Data are presented as number (percent).

*, ten thousand Korean won.

P-value by Chi-square test.

EC, emergency contraception; USD, US dollar.

**Table 2** shows a comparison of the most important sources of information about contraception for users and non-users of EC. Healthcare provider was the most important source in about 25% of women. The order of the most important sources of information was similar between EC users and non-users.

**Table 2:** Most important source of information about contraception.

	EC use (N = 1,011)		*P*-value
	No (N = 550)	Yes (N = 461)	
Healthcare provider (N=264)	150 (27.3)	114 (24.7)	0.516
Social media (N=244)	129 (23.5)	115 (24.9)	
Acquaintances (N=229)	124 (22.5)	105 (22.8)	
Mass media (N=211)	119 (21.6)	92 (20.0)	
Sex partner (N=54)	23 (4.2)	31 (6.7)	
Others (N=9)	5 (0.9)	4 (0.9)	

Data are presented as number (percent).

P-value by Chi-square.

EC, emergency contraception.

**Figure 1:**
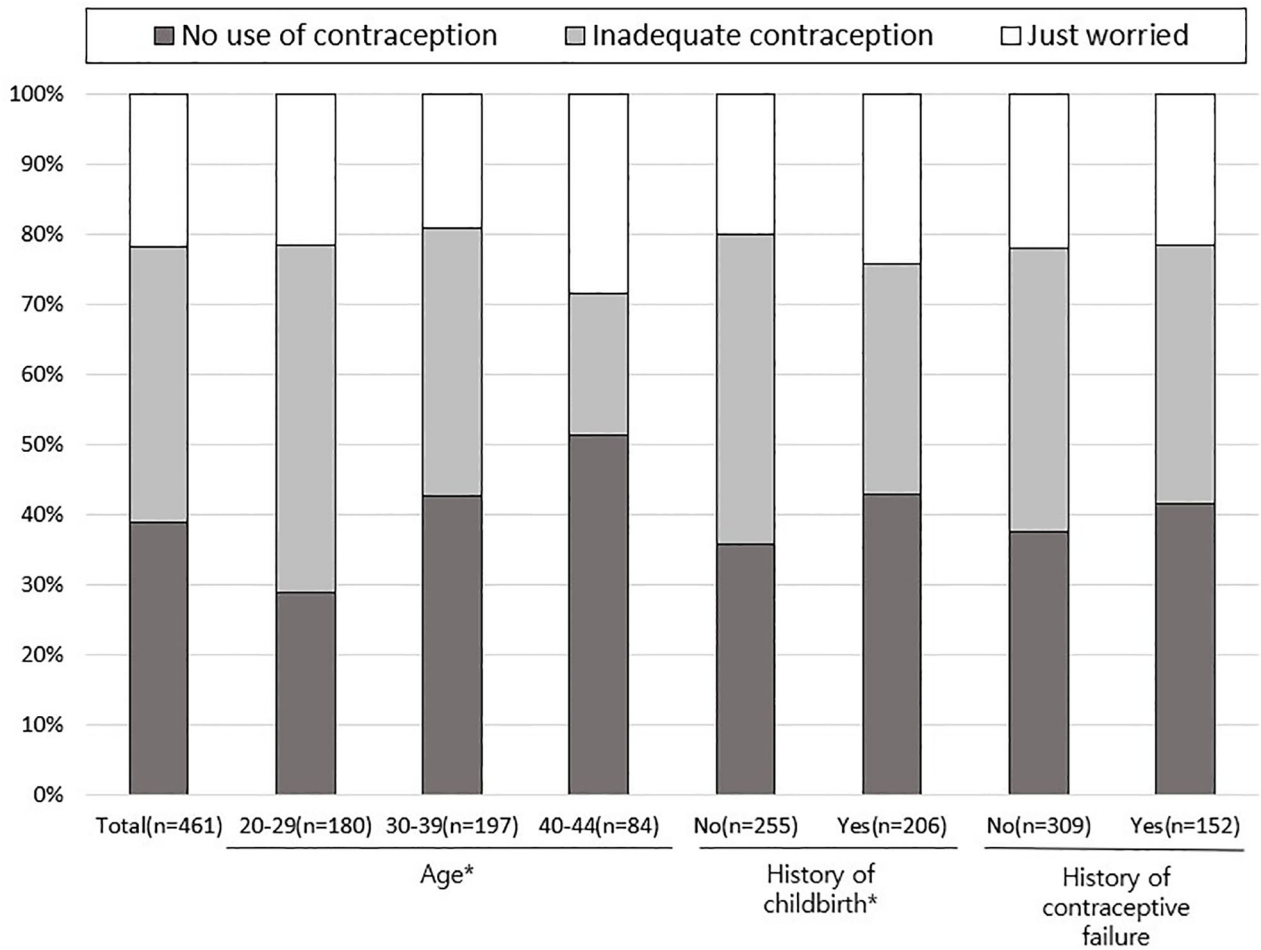
Reasons for using emergency contraception. ^*^ *P <*0.05 by Chi-square test.

**Figure 1** illustrates the reasons for EC use in the 461 users. The reasons for EC use were significantly different between women aged 40-44 years and 20-29 (P <0.001) and 30-39 years (P = 0.011). Among younger women, the proportion of women who used EC due to inadequate contraception (incorrect use of any contraceptive method such as missing birth control pills, tearing of condom or miscalculation of fertile period during sexual intercourse) was significantly higher (49.4% in 20–29, 38.1% in 30–39, and 28.6% in 40–44 years; P <0.001). In contrast, the proportions of women who used EC due to having not used contraception increased with age (28.9% in 20–29, 42.6% in 30–39, and 51.2% in 40–44 years; P <0.001). Furthermore, the proportion of women who used EC because they had not used contraception during sexual intercourse was significantly higher in women who had a history of childbirth (42.7% vs. 35.7%; P = 0.047). However, no difference was found according to history of contraceptive failure.

**Figure 2:**
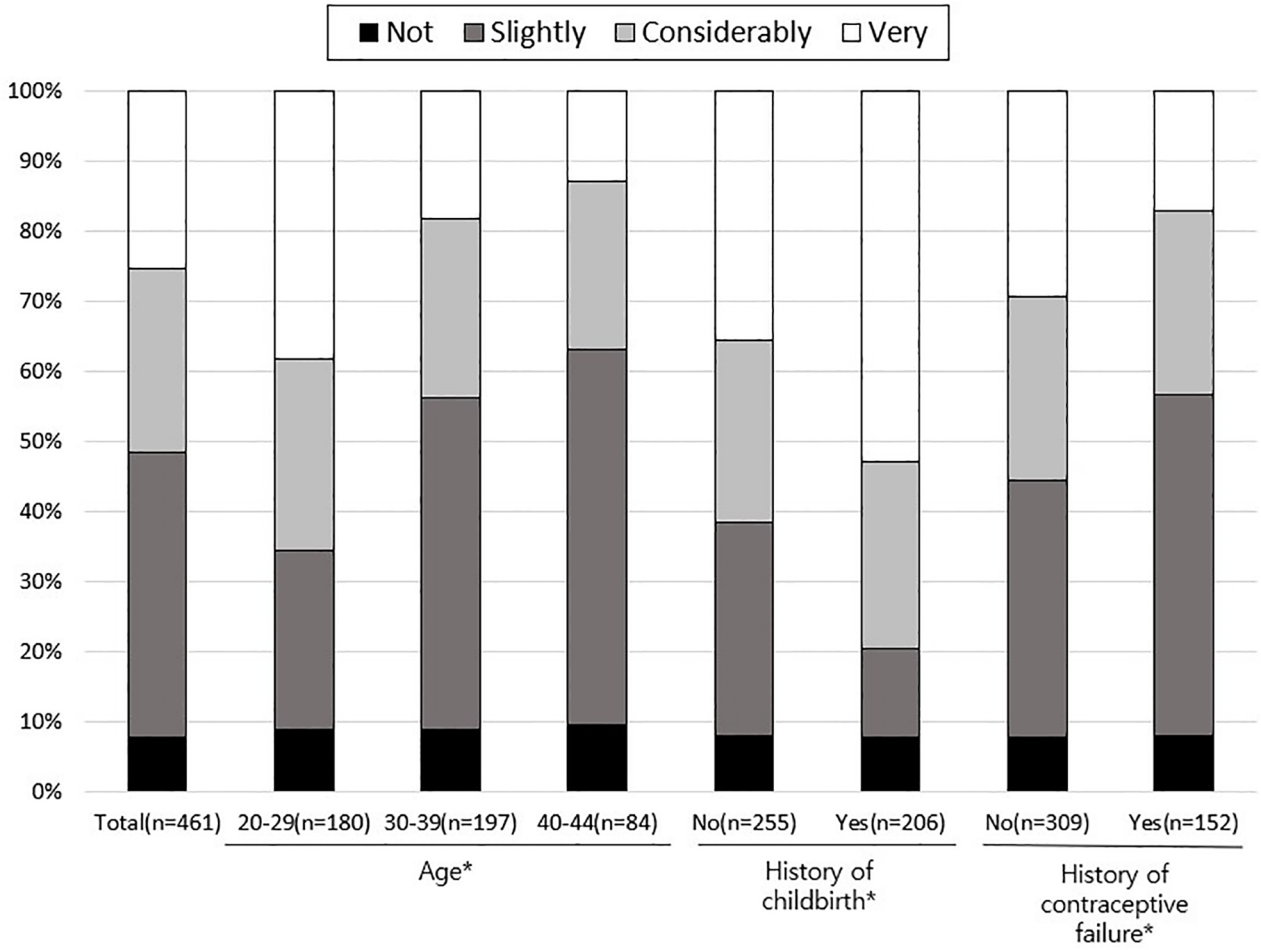
Anxiety at the time of using emergency contraception. ^*^ *P <*0.05 by Chi-square test.

The anxiety of women at the time of EC use is shown in **Figure 2**. Overall, the proportion of women who did not feel anxious was only 7.8% in EC users, whereas more than 50% reported feeling seriously concerned (considerably anxious = 26.2%, very anxious =25.4%). In EC users, the level of anxiety was significantly different between women aged 20-29 years and 30-39 (P <0.001) and 40-44 years (P = 0.001). The proportion of women with anxiety was significantly higher in younger age groups (38.3% in 20–29, 18.8% in 30–39, and 13.1% in 40–44 years; P <0.001). Additionally, the proportions of women with anxiety were lower among those with a history of childbirth (P <0.001) and those with history of contraceptive failure (P = 0.004).

**Figure 3:**
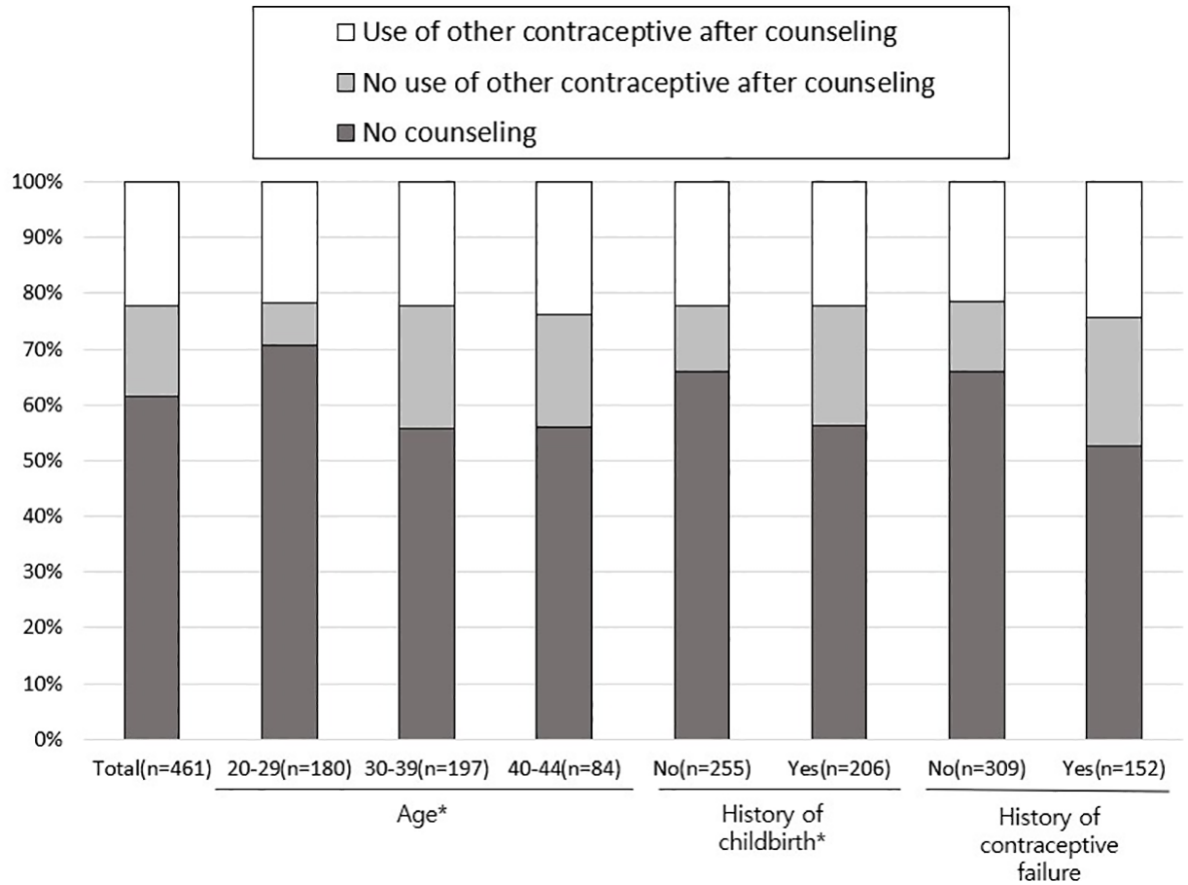
Counseling for additional contraception at the time of using emergency contraception. ^*^ *P <*0.05 by Chi-square test.

Experience with counseling for contraception at the time of EC use is presented in **Figure 3**. Overall, 61.6% of women did not get contraception counseling when they received an EC prescription from their healthcare provider. Only 22.3% were able to start use of another non-emergency contraceptive method after counseling. In women aged 20–29 years, the proportion who had no contraception counseling was significantly higher than in women aged 30-39 (P <0.001) and 40-44 years (P <0.001). Additionally, the proportion of women who sought no contraception counseling was higher in women who did not have a history of childbirth (P = 0.026).

## Discussion

In this nationwide online survey evaluating current EC use in reproductive-aged Korean women, the proportion of women who ever experienced EC use was almost 50%, which was similar to a population-based survey in Brazil that found approximately half of women aged 15–44 had used EC at least once (11). However, this result is higher than those from a recent European study reporting that 37% of reproductive-aged (18–49 years) women experienced EC use (10) and other studies in European and American countries that reported a prevalence of 30% or less (12–15). Although the prevalence of EC use differs according to age, target population, region, time, and medical environments such as policy related to doctor prescriptions, a common feature is that EC use prevalence continues to increase worldwide alongside increasingly positive attitudes toward EC (16).

Our results are consistent with other studies reporting that women younger than 35 years were more likely to use EC (11, 17). However, education level was not different in users and non-users in our study, in contrast with other studies that found higher education level to be associated with higher EC use (11, 13, 18). This difference might result from much higher education level of participants in our study (~90% of respondents had 12 or more years of schooling). Indeed, the percentage of women with years of schooling ≥ 13 was about 35% (11) and that with a bachelor’s degree or higher was 28% (18) in EC users in other studies. Additionally, no association between income and EC use was found in our study, which was also different from a previous study (12). As cost is a well-known barrier to EC use, the generally higher income level in our participants and the relatively inexpensive cost of EC in Korea could account for this difference.

Healthcare provider was the most important source of information about contraception in this study, in line with another survey of young women reporting that clinicians were the most preferred and trusted source of contraceptive information among >80% of respondents (19). However, the proportion of healthcare provider as the most important source of information was only about 25%, and young women usually acquired information from advertisements or word-of-mouth in the present study. This finding suggests an unmet need for healthcare experts to enhance delivery of reliable and objective information. However, the most effective mode of delivery for information has not been established, although various methods such as the internet, mobile phones, or videos have been evaluated (20).

The proportion of women who used EC due to inadequate contraception was higher among younger respondents. This is consistent with previous studies that found the proportion of young women not using adequate contraception was high (21, 22). Young women may not be familiar with the correct and consistent use of effective contraceptive methods. Undoubtedly, inadequate contraceptive use ultimately leads to repeated EC use, and recent studies reported that 46%–67.4% of EC users used EC more than once (10, 11). Indeed, 79% of physicians responded to a survey that the most important factor for preventing emergency contraceptive pill abuse was contraceptive education (23). In the present study, many young women felt considerably anxious at the time of EC use, and this result is in contrast that about 70% of women have responded that they had sufficient knowledge about EC in the Thinking About Life with Contraception in Korean Women study (10). Nevertheless, over two-thirds of young women did not receive any counseling regarding contraception during their visit for an EC prescription. Taken together, the reasons for use, level of anxiety, and experience with counseling for contraception during EC use observed in the present study indicate the importance of well-organized and practical education and counseling to introduce or establish reliable and long-term contraceptive use in young EC users. According to a previous study, 73% of women responded that they would consider long-acting reversible contraception that is reliable and safe if they had more comprehensive information (9).

Considering long-term contraception and future fertility, the importance of a strategy that enhances appropriate contraception use cannot be overemphasized in young women. Women who had experienced EC use are at high risk of unintended pregnancy in the near future, because many do not use an appropriate contraceptive method after EC use (24, 25). Information about the efficacy and safety of various contraceptive methods can be provided via preferred communication channels or during individualized counseling sessions linked to healthcare visits for an EC prescription. Friendly social or medical environments for discussing contraceptive use could also be helpful for young women.

Meanwhile, the contraceptive needs of older women also should not be underestimated. In the current study, as women age, they are more likely to use EC due to lack of a regular contraceptive method, especially women aged 40–44 years. Because unwanted pregnancies among women in their late 30s and early 40s who have completed their desired childbearing are not uncommon and often lead to abortion, appropriate contraception is important in this age group. It has been reported that the percentage of pregnancies that are unintended is over 30% in women aged 35 or more, and that the percentage of unintended pregnancies that end in abortion is higher in women aged 35 or more than in any other younger age groups (1).

This study has several strengths. First, we utilized data from a nationally representative survey for which participants were randomly recruited and weighted to reflect Korean census estimates. Past studies regarding EC use were mainly conducted among university students in Korea (7, 8). Second, only women who had visited a clinic in the previous six months for contraception counseling were included, which enabled us to assess current EC use in real clinical practice. In contrast, in other studies in Korea, 42% (7) and 62.6% (8) of women participants had never experienced sexual intercourse, and awareness or attitude toward EC, rather than real use, were addressed.

However, this study also has limitations. First, online surveys have several potential biases. Specifically, information regarding EC use is self-reported and is subject to recall bias. Second, this study utilized data regarding EC use from another study (10) that was designed to address general contraception, not EC specifically, in Korean women aged 20–44 years. Therefore, we did not have detailed information regarding demographics or history of marriage, sexual health and contraception related to EC use. Additionally, adolescents, who may use EC with less guidance and more misunderstandings (26, 27), were not included.

In conclusion, this study shows that young women are more likely to use EC due to inadequate contraception and are often seriously concerned about their EC use, but they also have fewer opportunities for counseling during EC use, compared with older women. These findings offer insights for developing and improving individualized strategies for appropriate contraception in young EC users in Korea.

## Data availability statement

The original contributions presented in the study are included in the article/supplementary material, further inquiries can be directed to the corresponding author/s.

## Ethics statement

The studies involving human participants were reviewed and approved by Samsung Medical Center. The patients/participants provided their written informed consent to participate in this study.

## Author contributions

Conceptualization: D-YL. Data curation: YL, D-YL. Formal analysis: YL, SK, D-YL. Investigation: YL, SK, D-YL, DC. Methodology: YL, D-YL. Project administration: D-YL, DC. Supervision: D-YL. Validation: D-YL. Visualization: YL, D-YL. Writing - original draft: YL, D-YL. All authors contributed to the article and approved the submitted version.
